# Supplementary material for: Limits to load-lifting performance in a passerine bird: the effects of intraspecific variation in morphological and kinematic parameters
Source: PeerJ. 2019 Nov 13;7:e8048. doi: 10.7717/peerj.8048 (PMC6858814; doi:10.7717/peerj.8048)
Supplement: Supplemental Information 1 [file peerj-07-8048-s001.docx]

**Table S1. Statistical effects of morphological variables in the best structural equation model explaining variation in total lifted load by Eurasian tree sparrows (*Passer* *montanus*).**

| **Regression** | **Variable** | **Estimate** | **Se** | ***z*** | ***p* value** |
| --- | --- | --- | --- | --- | --- |
| **Total lifted load** | Bill length | -0.257 | 0.080 | -3.229 | **0.001** |
|  | Stomach mass | 0.231 | 0.079 | 2.911 | **0.004** |
|  | Flight muscle mass | 0.622 | 0.086 | 7.235 | **<0.001** |
|  | Wing area | 0.194 | 0.082 | 2.353 | **0.019** |

**Table S2. Covariances among variables in the best structural equation model explaining variation in total lifted load by Eurasian tree sparrows (*Passer* *montanus*).**

| **Covariances** | | **Estimate** | **Se** | ***z*** | ***p* value** |
| --- | --- | --- | --- | --- | --- |
| **Bill length ~ ~** | Stomach mass | 0.092 | 0.117 | 0.791 | 0.429 |
|  | Flight muscle mass | 0.253 | 0.120 | 2.105 | **0.035** |
|  | Wing area | 0.127 | 0.117 | 1.086 | 0.278 |
| **Stomach mass ~ ~** | Flight muscle mass | 0.202 | 0.119 | 1.699 | 0.089 |
|  | Wing area | -0.045 | 0.116 | -0.383 | 0.702 |
| **Flight muscle mass ~ ~** | Wing area | 0.336 | 0.123 | 2.735 | **0.006** |
